# Supplementary material for: Peptide clustering enhances large-scale analyses and reveals proteolytic signatures in mass spectrometry data
Source: Nat Commun. 2024 Aug 20;15:7128. doi: 10.1038/s41467-024-51589-y (PMC11336174; doi:10.1038/s41467-024-51589-y)
Supplement: Supplementary file 1 — Supplementary Information [file 41467_2024_51589_MOESM1_ESM.pdf]

Supplementary Figures

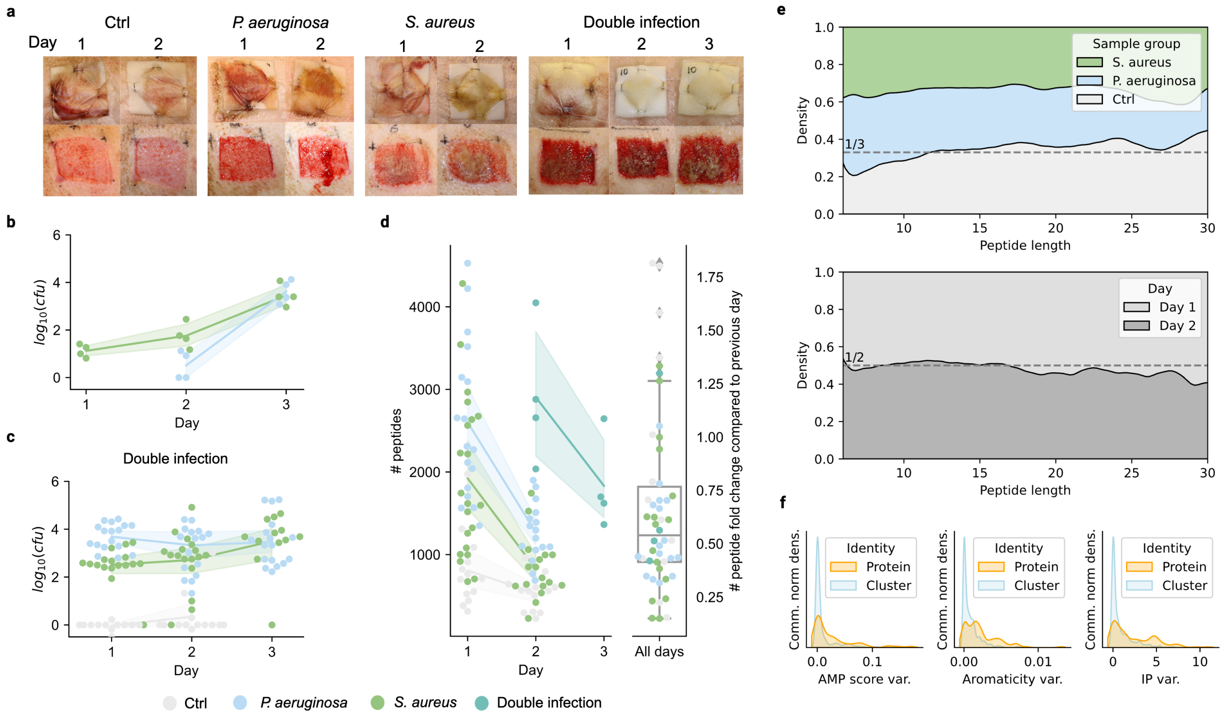

Supplementary Fig. 1 The porcine wound fluid peptidome. a Representative pictures of the porcine wounds with and without dressing over the sampling period. Wounds were infected on day 0. The double infected wounds were infected with *P. aeruginosa* on day 1. **b** log_{10}CFU over the three days in the single infections and control wounds. **c** log_{10}CFU over the three days in the double-infected wounds. In **b** and **c** the line shows the mean trend and the error bars ±1SD. **d** Number of peptides detected over the timespan. The right panel shows the fold change of the number of peptides between day n and $n-1$. Every sample is shown as a scatter, alongside a line for mean values. For the means, ±1 SD is shown as error-bands. **e** Peptide length profiles when comparing infection type and days. **f** Inter-cluster and inter-protein variance of AMP score, aromaticity, and isoelectric point of peptides.

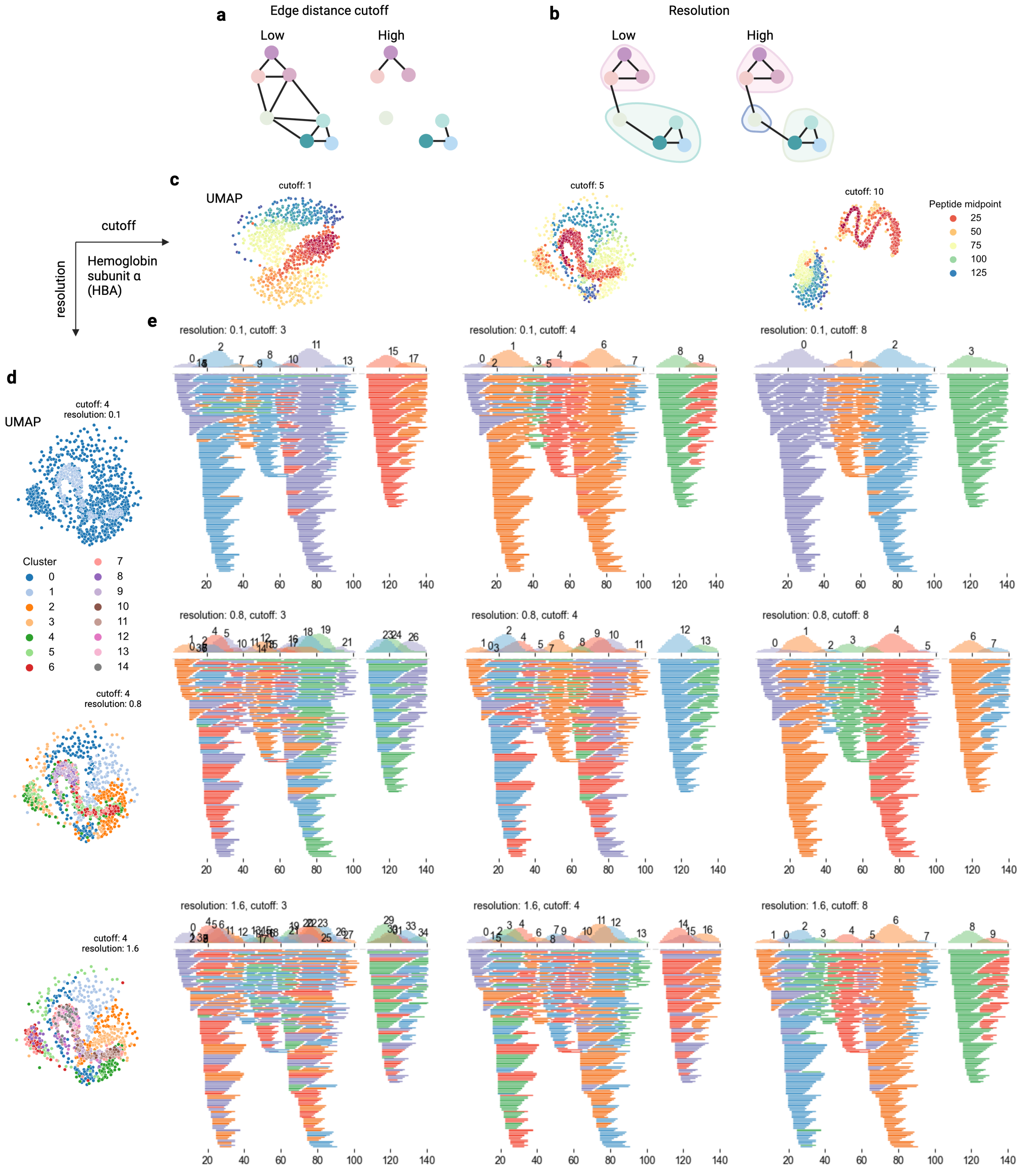

Supplementary Fig. 2 The impact of parameters on the clustering of peptidomes. a An illustration showcasing the impact of the edge distance cutoff used in peptide network creation. **b** An illustration showcasing the impact of the resolution parameter in peptide cluster partitioning with the Leiden algorithm. **c** The networks when varying the cutoff (1,5,10) were projected to 2 dimensions using uniform manifold approximation and projection (UMAP). Each node is a peptide in Hemoglobin subunit α (HBA) and the node colors reflect the peptide midpoints. **d** Peptide networks for HBA were created with a cutoff of 4 and then partitioned with a varying resolution parameter (0.1, 0.8, 1.6). The nodes are colored based on their cluster designation. **e** Peptide projections when varying the resolution (y-direction) and cutoff (x-direction). The peptides are colored based on their cluster designation. The middle panel shows a resolution of 0.8 and

a cutoff of 4, which were the parameter values used in the study.

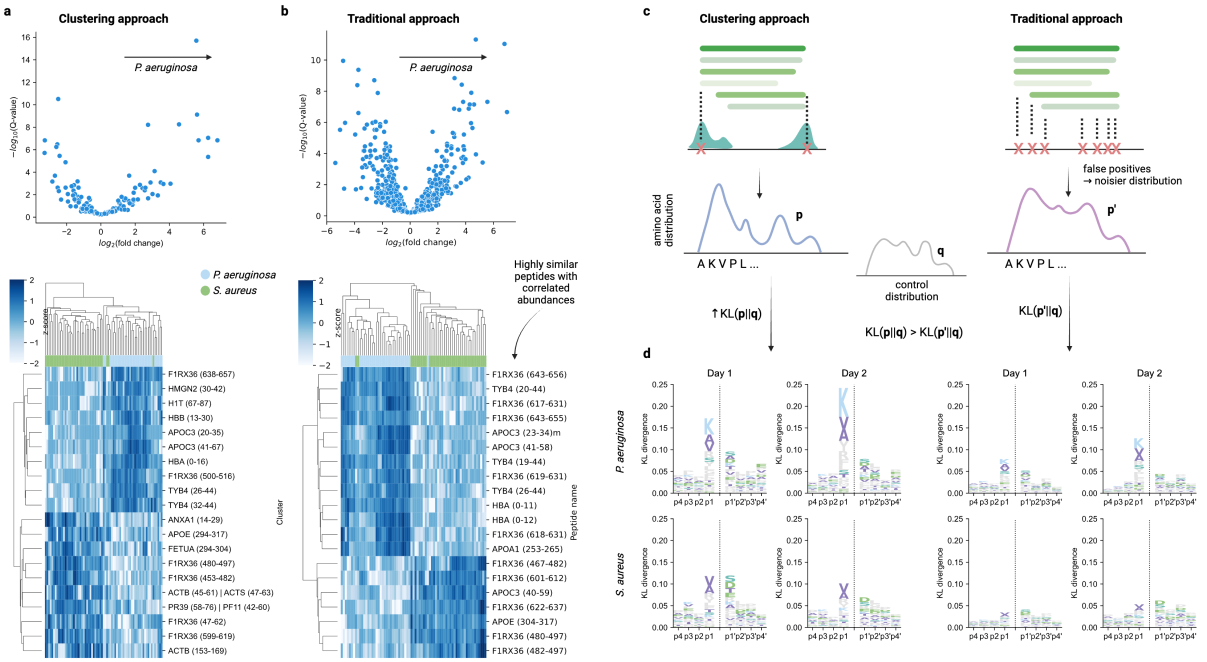

Supplementary Fig. 3 Comparison between clustering and traditional analyses. **a** The clustering approach entails creating clusters and quantifying them. Here we quantified the clusters with the top 3 most differentially abundant peptides. The top panel shows a volcanoplot. The Q-value is calculated by computing a p-value using linear regression followed by Benjamini-Hochberg correction for multiple hypothesis testing. The bottom panel shows a clustermap of the top 20 clusters by Q-value. **b** Similar to **a** but with a traditional peptidomics approach. In the clustermap, it is apparent that many peptides are highly similar and highly correlated. m denotes modification. **c** An illustration of the benefits of using clusters for cut site identification. The sharper distribution is a result of removing the false positive cut sites introduced by exoproteases and leads to a larger KL divergence. **d** Logoplots showcasing the p4-p4' cut sites when using a clustering approach (left) vs. a traditional approach (right).

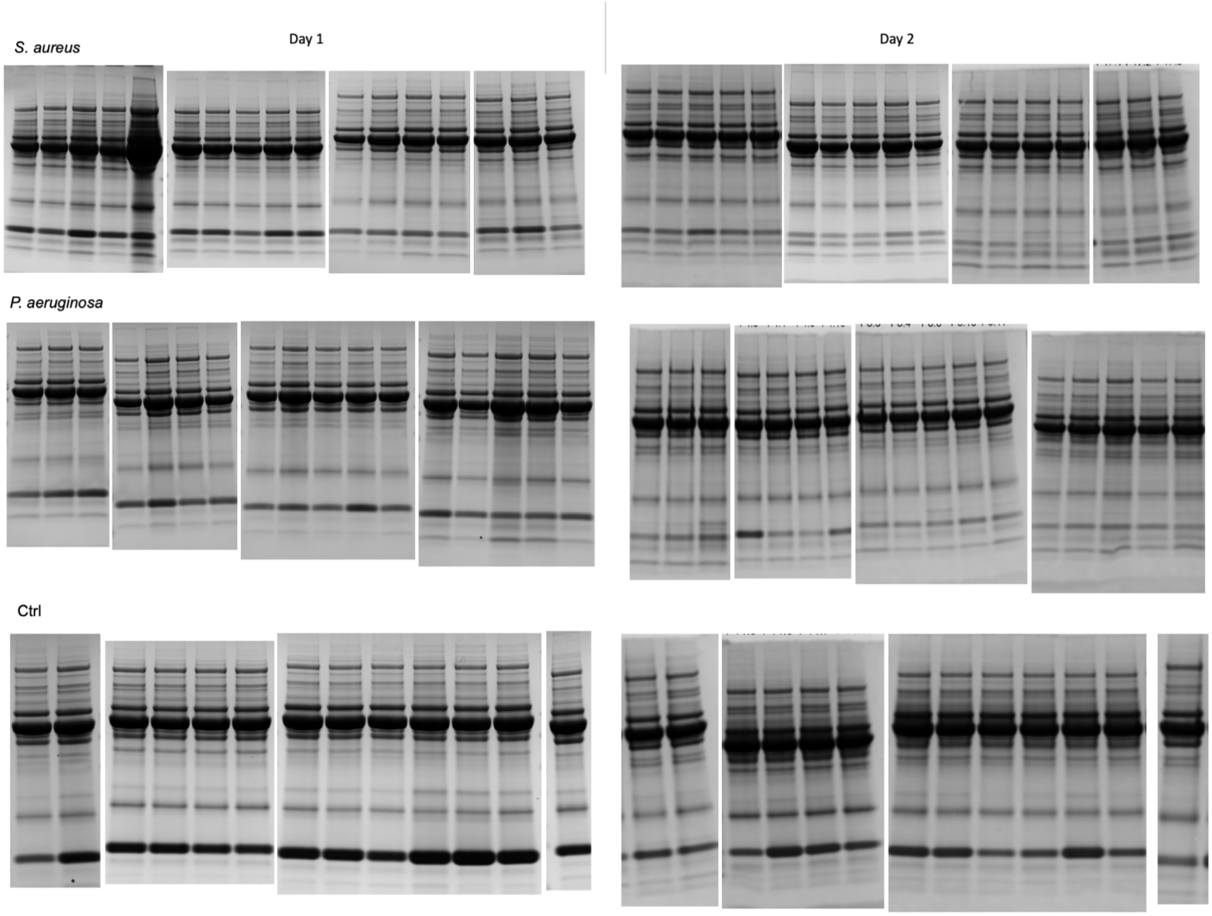

Supplementary Fig. 4 SDS-PAGE for sample types. SDS-PAGE on the wound fluid of all samples on day 1 (left) and day 2 (right). The upper row shows the sample s infected by *S. aureus*, the middle by *P. aeruginosa* and the lower the uninfected control samples. Uncropped gels are available in the Source Data. SDS was performed once for each sample.

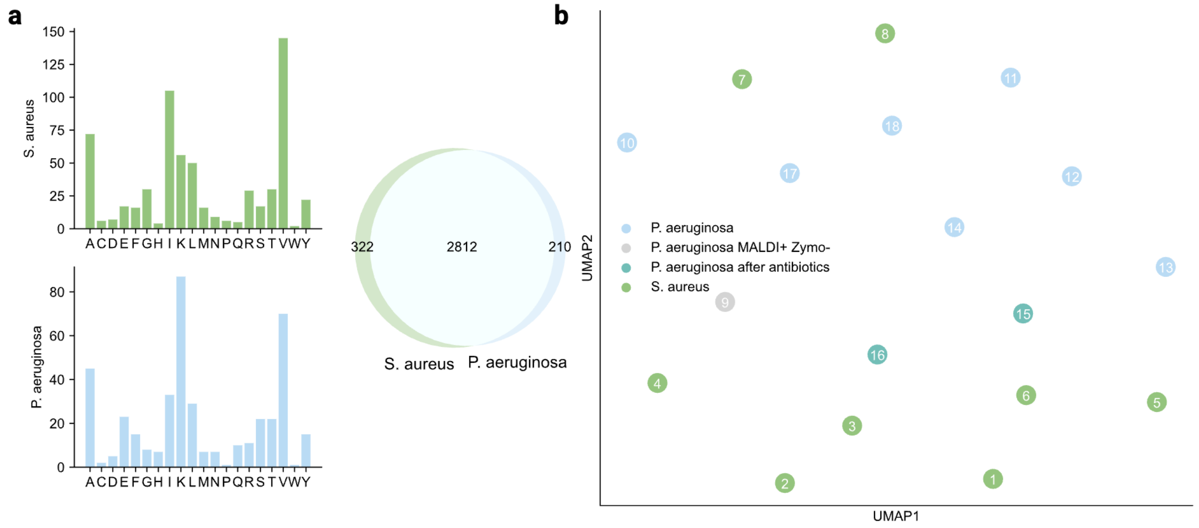

Supplementary Fig. 5 Human wound fluid stratification. **a** Amino acid profile for the amino acids at the p1 position for the unique clusters when stratifying the samples on the species of the primary colonizer. **b** UMAP of the quantified peptide clusters colored based on results from MALDI and zymograms.

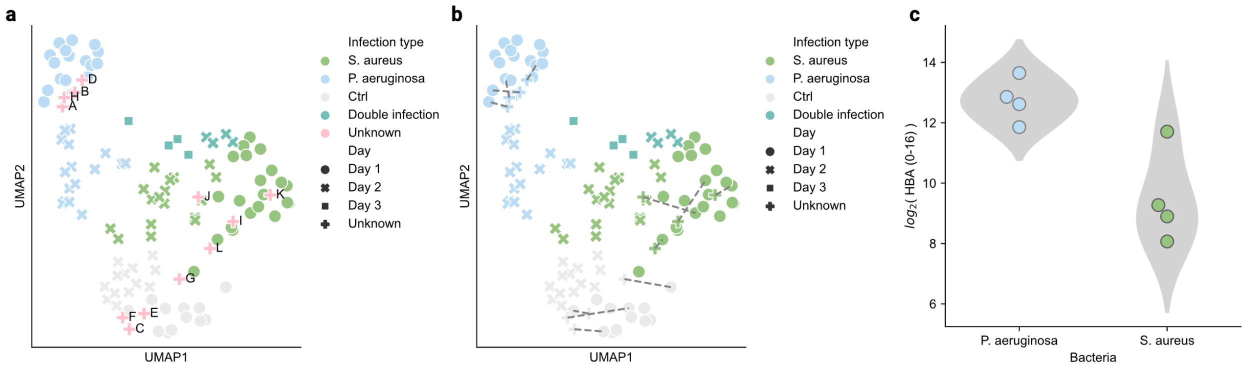

Supplementary Fig. 6 Blinded replicate analysis. **a** A UMAP visualization of the original dataset with the inclusion of new replicate samples, prior to revealing the key. **b** The same UMAP as depicted in **(a)** after revealing the key. Lines are drawn to connect each replicate sample with its corresponding sample in the original dataset. **c** Violin plots illustrating the intensity distribution of the HBA 0-16 cluster in replicate samples sourced from wounds infected by *P. aeruginosa* and *S. aureus respectively*.

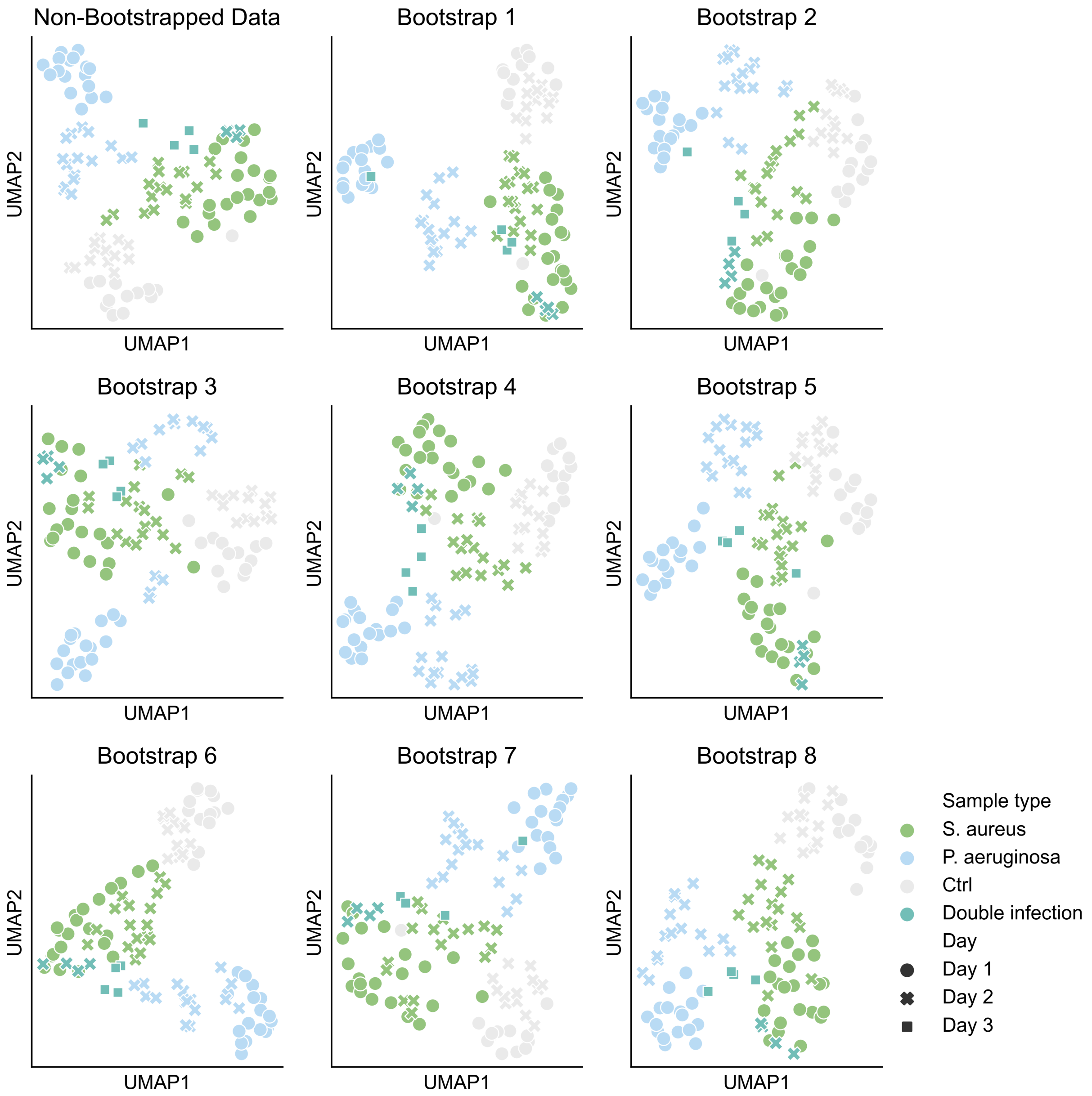

Supplementary Fig. 7 UMAP projections of bootstrapped and down-sampled peptide clusters. The original feature matrix contains the scaled quantities of 743 peptide clusters. During the bootstrap, 371 clusters are picked randomly and a UMAP is fitted to the subsampled data. The reduced data is visualized.

Supplementary Tables

Supplementary Table 1. Sample identity specifications for porcine samples.

Sample ID	Infection type	Day	Pig ID	Wound ID
Sample 1 Day 1	*S. aureus*	Day 1	2	1
Sample 1 Day 2	*S. aureus*	Day 2	2	1
Sample 2 Day 1	*S. aureus*	Day 1	2	2
Sample 2 Day 2	*S. aureus*	Day 2	2	2
Sample 3 Day 1	*S. aureus*	Day 1	2	3

Sample ID	Infection type	Day	Pig ID	Wound ID
Sample 3 Day 2	*S. aureus*	Day 2	2	3
Sample 4 Day 1	*S. aureus*	Day 1	2	9
Sample 4 Day 2	*S. aureus*	Day 2	2	9
Sample 5 Day 1	*S. aureus*	Day 1	2	10
Sample 5 Day 2	*S. aureus*	Day 2	2	10
Sample 6 Day 1	Accidental double infection	Day 1	5	3
Sample 6 Day 2	Accidental double infection	Day 2	5	3
Sample 7 Day 1	Accidental double infection	Day 1	5	4
Sample 7 Day 2	Accidental double infection	Day 2	5	4
Sample 8 Day 1	Accidental double infection	Day 1	5	6
Sample 8 Day 2	Accidental double infection	Day 2	5	6
Sample 9 Day 1	Accidental double infection	Day 1	5	10
Sample 9 Day 2	Accidental double infection	Day 2	5	10
Sample 10 Day 1	*S. aureus*	Day 1	6	3
Sample 10 Day 2	*S. aureus*	Day 2	6	3
Sample 11 Day 1	*S. aureus*	Day 1	6	4
Sample 11 Day 2	*S. aureus*	Day 2	6	4
Sample 12 Day 1	*S. aureus*	Day 1	6	6
Sample 12 Day 2	*S. aureus*	Day 2	6	6
Sample 13 Day 1	*S. aureus*	Day 1	6	11
Sample 13 Day 2	*S. aureus*	Day 2	6	11
Sample 14 Day 1	*S. aureus*	Day 1	17	1
Sample 14 Day 2	*S. aureus*	Day 2	17	1
Sample 15 Day 1	*S. aureus*	Day 1	17	2
Sample 15 Day 2	*S. aureus*	Day 2	17	2
Sample 16 Day 1	*S. aureus*	Day 1	17	5
Sample 16 Day 2	*S. aureus*	Day 2	17	5
Sample 17 Day 1	*P. aeruginosa*	Day 1	3	1
Sample 17 Day 2	*P. aeruginosa*	Day 2	3	1
Sample 18 Day 1	*P. aeruginosa*	Day 1	3	2
Sample 18 Day 2	*P. aeruginosa*	Day 2	3	2
Sample 19 Day 1	*P. aeruginosa*	Day 1	3	3
Sample 19 Day 2	*P. aeruginosa*	Day 2	3	3
Sample 20 Day 1	*P. aeruginosa*	Day 1	4	3
Sample 20 Day 2	*P. aeruginosa*	Day 2	4	3
Sample 21 Day 1	*P. aeruginosa*	Day 1	4	4
Sample 21 Day 2	*P. aeruginosa*	Day 2	4	4
Sample 22 Day 1	*P. aeruginosa*	Day 1	4	6
Sample 22 Day 2	*P. aeruginosa*	Day 2	4	6
Sample 23 Day 1	*P. aeruginosa*	Day 1	4	10
Sample 23 Day 2	*P. aeruginosa*	Day 2	4	10
Sample 24 Day 1	*P. aeruginosa*	Day 1	8	3
Sample 24 Day 2	*P. aeruginosa*	Day 2	8	3
Sample 25 Day 1	*P. aeruginosa*	Day 1	8	4
Sample 25 Day 2	*P. aeruginosa*	Day 2	8	4
Sample 26 Day 1	*P. aeruginosa*	Day 1	8	6
Sample 26 Day 2	*P. aeruginosa*	Day 2	8	6
Sample 27 Day 1	*P. aeruginosa*	Day 1	8	10
Sample 27 Day 2	*P. aeruginosa*	Day 2	8	10
Sample 28 Day 1	*P. aeruginosa*	Day 1	8	11
Sample 28 Day 2	*P. aeruginosa*	Day 2	8	11
Sample 29 Day 1	*P. aeruginosa*	Day 1	11	3

Sample ID	Infection type	Day	Pig ID	Wound ID
Sample 30 Day 1	*P. aeruginosa*	Day 1	11	4
Sample 30 Day 2	*P. aeruginosa*	Day 2	11	4
Sample 31 Day 1	*P. aeruginosa*	Day 1	11	6
Sample 31 Day 2	*P. aeruginosa*	Day 2	11	6
Sample 32 Day 1	*P. aeruginosa*	Day 1	11	7
Sample 32 Day 2	*P. aeruginosa*	Day 2	11	7
Sample 33 Day 1	*P. aeruginosa*	Day 1	11	8
Sample 33 Day 2	*P. aeruginosa*	Day 2	11	8
Sample 34 Day 1	Ctrl	Day 1	10	6
Sample 34 Day 2	Ctrl	Day 2	10	6
Sample 35 Day 1	Ctrl	Day 1	10	11
Sample 35 Day 2	Ctrl	Day 2	10	11
Sample 36 Day 1	Ctrl	Day 1	14	3
Sample 36 Day 2	Ctrl	Day 2	14	3
Sample 37 Day 1	Ctrl	Day 1	14	6
Sample 37 Day 2	Ctrl	Day 2	14	6
Sample 38 Day 1	Ctrl	Day 1	14	7
Sample 38 Day 2	Ctrl	Day 2	14	7
Sample 39 Day 1	Ctrl	Day 1	14	11
Sample 39 Day 2	Ctrl	Day 2	14	11
Sample 40 Day 1	Ctrl	Day 1	16	3
Sample 40 Day 2	Ctrl	Day 2	16	3
Sample 41 Day 1	Ctrl	Day 1	16	4
Sample 41 Day 2	Ctrl	Day 2	16	4
Sample 42 Day 1	Ctrl	Day 1	16	6
Sample 42 Day 2	Ctrl	Day 2	16	6
Sample 43 Day 1	Ctrl	Day 1	16	7
Sample 43 Day 2	Ctrl	Day 2	16	7
Sample 44 Day 1	Ctrl	Day 1	16	8
Sample 44 Day 2	Ctrl	Day 2	16	8
Sample 45 Day 1	Ctrl	Day 1	16	11
Sample 45 Day 2	Ctrl	Day 2	16	11
Sample 46 Day 1	Ctrl	Day 1	24	1
Sample 46 Day 2	Ctrl	Day 2	24	1
Sample 54 Day 1	*S. aureus*	Day 1	21	1
Sample 54 Day 2	Double infection	Day 2	21	1
Sample 54 Day 3	Double infection	Day 3	21	1
Sample 55 Day 1	*S. aureus*	Day 1	21	2
Sample 55 Day 2	Double infection	Day 2	21	2
Sample 55 Day 3	Double infection	Day 3	21	2
Sample 56 Day 1	*S. aureus*	Day 1	21	5
Sample 56 Day 2	Double infection	Day 2	21	5
Sample 56 Day 3	Double infection	Day 3	21	5
Sample 57 Day 1	*S. aureus*	Day 1	21	9
Sample 57 Day 2	Double infection	Day 2	21	9
Sample 57 Day 3	Double infection	Day 3	21	9
Sample 66 Day 1	*S. aureus*	Day 1	12	1
Sample 66 Day 2	*S. aureus*	Day 2	12	1
Sample 67 Day 1	*S. aureus*	Day 1	12	2
Sample 67 Day 2	*S. aureus*	Day 2	12	2
Sample 68 Day 1	*S. aureus*	Day 1	12	5
Sample 68 Day 2	*S. aureus*	Day 2	12	5

Sample ID	Infection type	Day	Pig ID	Wound ID
Sample 69 Day 1	_S. aureus_	Day 1	12	7
Sample 69 Day 2	_S. aureus_	Day 2	12	7
Sample 70 Day 1	_S. aureus_	Day 1	12	8
Sample 70 Day 2	_S. aureus_	Day 2	12	8

Supplementary Notes 6. Description of human chronic leg ulcer samples

Out of the 18 samples, 10 showed growth of _P. aeruginosa_. These samples also often contained other bacteria, such as _E. faecalis_, **S. lugdunensis, Corynebacterium sp._,_ S. aureus_, and_ S. agalactiae_. The other 8 samples primarily contained_ S. aureus_. Sample 9 contained_ P. aeruginosa* but did not show a _P. aerguinosa_-like profile on the zymogram, and is therefore labeled “P. aeruginosa MALDI+ Zymo-”. One patient was given antibiotics against a _P. aeruginosa_-infection, and two samples derived from this patient are therefore labeled as “P. aeruginosa after antibiotics”.

Supplementary Table 2. Sample identity specifications for human samples. Bacterial identification by MALDI was not performed on samples 17 and 18.

Sample	Patient	Day	Wound	NE group	Zymogram	Bacteria
1	2101	3	1	Low	_S. aureus_	_S. aureus_, _E. faecalis_, _S. lugdunensis_
2	2101	3	2	Low	_S. aureus_	_S. aureus_, _E. faecalis_, _S. lugdunensis_
3	2101	8	1	Low	_S. aureus_	_S. aureus_, _E. faecalis_, _Corynebacterium sp._
4	2101	8	2	Low	_S. aureus_	_S. aureus_, _E. faecalis_, _S. lugdunensis_, _Corynebacterium sp._
5	2104	3	1	Low	_S. aureus_	_S. aureus_
6	2104	3	2	Low	_S. aureus_	_S. aureus_
7	2104	8	1	High	_S. aureus_	_S. aureus_
8	2104	8	2	High	_S. aureus_	_S. aureus_
9	2103	3	1	Low	_S. aureus_	_P. aeruginosa_, _S. aureus_, _Corynebacterium sp._
10	2103	8	1	High	_P. aeruginosa_	_S. aureus P. aeruginosa E. faecalis Corynebacterium sp. P. rettger_

Sample	Patient	Day	Wound	NE group	Zymogram	Bacteria
11	2105	15	2	Low	*P. aeruginosa*	*S. aureus P. aeruginosa Corynebacterium sp. S. agalactiae*
12	2105	17	2	Medium	*P. aeruginosa*	-
13	2105	22	2	Medium	*P. aeruginosa*	*S. aureus*, *P. aeruginosa*, Corynebacteriaum sp., *S. agalactiae*, *A. xylosoxidans*
14	2105	24	2	Medium	*P. aeruginosa*	-
15	2105	29	1	Medium	*P. aeruginosa*	*P. aeruginosa*, *Corynebacterium sp.*, *A. xylosoxidans*
16	2105	31	2	Medium	*P. aeruginosa*	*P. aeruginosa*, *Corynebacterium sp.*
17	2105	3	1	High	*P. aeruginosa*	-
18	2105	3	1	High	*P. aeruginosa*	-

Supplementary Notes

Supplementary Notes 1. Characterization of the porcine wounds

The infected wounds developed clinical signs of infection, including erythema and visible bacterial biofilms (Supplementary Fig. 1a). The bacterial load in the single infections remained stable with around 10^3 CFU (Supplementary Fig. 1b). In the double infections, however, the CFU count of *S. aureus* increased when introducing *P. aeruginosa*, and the two species reached similar levels of colonization on day 3 (Supplementary Fig. 1c). The number of identified peptides decreased between days n and $n + 1$, with an average decrease of 34% (Supplementary Fig. 1d). Peptide lengths vary between 7 and 58 amino acids, with a mean length of 15.2 amino acids. Control samples contain a smaller fraction of short peptides (<11 amino acids) compared to infected samples, and peptide length decreases between days 1 and 2 (Supplementary Fig. 1e).

Previous work has shown that peptide properties are largely conserved in peptides with high sequence similarity (1,2). To investigate this and to motivate the use of peptide clusters as a functional entity, the antimicrobial tendencies of all peptides were predicted using a deep convolutional neural network (3). The network outputs a classification probability (antimicrobial prediction score, AMP score) for a given peptide sequence. The variance of the AMP score between peptides within a cluster was compared to the variance between all peptides in a protein as a benchmark. The inter-cluster variance is lower than the inter-protein variance of antimicrobial scores (Supplementary Fig. 1f). Isoelectric point and aromaticity showed similar distributions.

Supplementary Notes 2. The implication of parameter values in the clustering algorithm

The developed clustering methods entail two steps, both of which depend on parameters. Firstly, peptide networks with weighted edges are created. The goal during network creation is to connect peptides that are proximate in relation to their position on the protein backbone and have a significant degree of overlap. The distance between two peptides is computed by a metric with 3 terms. The first term computes the peptide overlap (high peptide overlap → short distance). A cutoff is then used to sever connections with a large distance, and it is this distance that we use as a parameter (Supplementary Fig. 2a,c).

The second computes the centroid distance (high centroid distance → large distance). The last considers the length-variability (high length variability → large distance). In the second step, the Leiden community detection algorithm is employed on the created networks, partitioning them further. The Leiden algorithm is dependent on a "resolution"-parameter. This parameter adjusts the coarseness of the clustering. Higher resolution leads to more communities (Supplementary Fig. 2b,d). The resulting clusters are dependent on both algorithms and tweaking parameters in one will affect the performance of the other.

Evaluating peptidomic clustering performance is difficult because there is no objective function that allows us to get a quantitative performance measure. To identify good hyperparameters, we visually inspected the resulting clusters when varying the cutoff and the resolution parameters. An example of such inspections can be seen in Supplementary Fig. 2e. We settled on using a resolution of 0.8 and a cutoff of 4, which provides the clustering that best captures the natural clustering of the peptidome.

Supplementary Notes 3. Comparing a traditional peptidomics approach with the clustering approach

To further showcase the utility of the clustering methodology, it was compared to a traditional peptidomics approach. Here, we perform a similar analysis pipeline on the porcine wound fluid data as the one presented in Fig. 7, on the dataset from Van et al. (4). For simplicity, we limit this analysis to the samples from singly infected wounds and seek to identify clusters and peptides that discriminate between the bacterial pathogens.

As can be seen in Supplementary Fig. 3a,b, a pure peptidomics approach identifies several peptides from the same region, which have highly correlated abundance profiles and are highly similar in sequence. This is not the case when analyzing peptide clusters.

In Supplementary Fig. 3c, we demonstrate the idea behind clustering as a means to improve the cut site identification. Each cluster is associated with two representative cut sites determined by taking the mode of the peptide positions (in cases of a tie, the longest peptide in the tie is used). These are assumed to be the positions that are cut by endoproteases. From a signal processing perspective, this is similar to reducing the noise in the signal, as the overwhelming false positives introduced by exoproteases are removed. This results in a sharper distribution, which results in a larger Kullback-Leibler (KL) divergence when compared against the control distribution. This is indeed the case, as a clustering approach gets a KL-divergence about 2-3 times the magnitude as compared to when using a traditional peptidomics approach (Supplementary Fig. 3d).

Supplementary Notes 4. Blinded re-analysis of 12 replicates

To ensure that our findings were reproducible, we re-analyzed a random subset of samples. These samples were chosen in a stratified but randomized manner from both singly infected samples and uninfected controls collected on day 1, with four samples selected from each group. The entire sample preparation pipeline and mass-spectrometry analysis were repeated but this time the sample annotations were blinded. We then analyzed the samples using the computational workflow and visualized the results using UMAPs to determine reproducibility. Since the analysis of the original samples, the mass spectrometry park has been upgraded from timsTOF pro to timsTOF HT.

The resulting UMAPs before and after revealing the key can be seen in Supplementary Fig. 6a and b respectively. The replicates group with the correct infection type and time point. To demonstrate that the specific signal identified in our original dataset is identifiable, we highlight that the cluster HBA 0-16 follows the same abundance profile in the replicate samples as previously identified (Supplementary Fig. 6c).

Supplementary Notes 5. Iterative down-sampled bootstrapping

To investigate how the missing values in our peptidomic clustering data affected the biological outcome, we bootstrapped and down-sampled our data to 50% of its original dimensionality and then reduced it further using UMAP. The results are shown in Supplementary Fig. 6, alongside the non-bootstrapped UMAP. If the number of missing values and irrelevant features greatly exceeded the number of informative features, bootstrapping and down-sampling would likely result in a high proportion of non-informative features. In such a case, the UMAP projection would not consistently show similar clustering patterns across different bootstrap iterations. However, our test showed that the data still clusters by timepoint and sample type, even after down-sampling. This indicates that our data is robust to the down-sampling process and the presence of missing values.

Supplementary References

1. Veldhuizen EJA, Schneider VAF, Agustiandari H, Dijk A van, Bokhoven JLMT, Bikker FJ, et al. Antimicrobial and immunomodulatory activities of PR-39 derived peptides. Cole AM, editor. PLoS ONE [Internet]. 2014 Apr;9(4):e95939. Available from: https://doi.org/10.1371/journal.pone.0095939

2. Hartman E, Wallblom K, Plas MJA van der, Petrlova J, Cai J, Saleh K, et al. Bioinformatic analysis of the wound peptidome reveals potential biomarkers and antimicrobial peptides. Frontiers in Immunology [Internet]. 2021 Feb;11. Available from: https://doi.org/10.3389/fimmu.2020.620707

3. Veltri D, Kamath U, Shehu A. Deep learning improves antimicrobial peptide recognition. Hancock J, editor. Bioinformatics [Internet]. 2018 Mar;34(16):2740–7. Available from: https://doi.org/10.1093/bioinformatics/bty179

4. Van JAD, Clotet-Freixas S, Zhou J, Batruch I, Sun C, Glogauer M, et al. Peptidomic analysis of urine from youths with early type 1 diabetes reveals novel bioactivity of uromodulin peptides in vitro. Molecular & Cellular Proteomics [Internet]. 2020 Mar;19(3):501–17. Available from: http://dx.doi.org/10.1074/mcp.RA119.001858
