# Supplementary material for: Peptide clustering enhances large-scale analyses and reveals proteolytic signatures in mass spectrometry data
Source: Nat Commun. 2024 Aug 20;15:7128. doi: 10.1038/s41467-024-51589-y (PMC11336174; doi:10.1038/s41467-024-51589-y)
Supplement: Supplementary file 2 — Reporting Summary [file 41467_2024_51589_MOESM2_ESM.pdf]

nature portfolio

Corresponding author(s): Erik Hartman

Last updated by author(s): Jul 13, 2024

# Reporting Summary

Nature Portfolio wishes to improve the reproducibility of the work that we publish. This form provides structure for consistency and transparency in reporting. For further information on Nature Portfolio policies, see our Editorial Policies and the Editorial Policy Checklist.

## Statistics

For all statistical analyses, confirm that the following items are present in the figure legend, table legend, main text, or Methods section.

| n/a | Confirmed | |
|---|---|---|
| ☐ | ☒ | The exact sample size (*n*) for each experimental group/condition, given as a discrete number and unit of measurement |
| ☐ | ☒ | A statement on whether measurements were taken from distinct samples or whether the same sample was measured repeatedly |
| ☐ | ☒ | The statistical test(s) used AND whether they are one- or two-sided<br>*Only common tests should be described solely by name; describe more complex techniques in the Methods section.* |
| ☐ | ☒ | A description of all covariates tested |
| ☐ | ☒ | A description of any assumptions or corrections, such as tests of normality and adjustment for multiple comparisons |
| ☐ | ☒ | A full description of the statistical parameters including central tendency (e.g. means) or other basic estimates (e.g. regression coefficient) AND variation (e.g. standard deviation) or associated estimates of uncertainty (e.g. confidence intervals) |
| ☐ | ☒ | For null hypothesis testing, the test statistic (e.g. *F*, *t*, *r*) with confidence intervals, effect sizes, degrees of freedom and *P* value noted<br>*Give P values as exact values whenever suitable.* |
| ☒ | ☐ | For Bayesian analysis, information on the choice of priors and Markov chain Monte Carlo settings |
| ☒ | ☐ | For hierarchical and complex designs, identification of the appropriate level for tests and full reporting of outcomes |
| ☐ | ☒ | Estimates of effect sizes (e.g. Cohen's *d*, Pearson's *r*), indicating how they were calculated |

*Our web collection on statistics for biologists contains articles on many of the points above.*

## Software and code

Policy information about availability of computer code

Data collection

After data-collection with tandem mass spectrometry, the raw data was searched with the commercial program PEAKS X to retrieve the peptide matrices.

Data analysis

Custom Python-code was developed for data analysis. A generalized package of the developed code has been deposited to an open GitHub repository under an MIT license: https://github.com/ErikHartman/pepnets

The data analysis was dependent on open-source Python packages available through pip, mainly dependent on the following packages
networkx 3.1,
leidenalg 0.10.1,
pandas 2.0.3,
matplotlib 3.7.2,
numpy 1.21.5,
seaborn 0.12.2,
igraph 0.10.6,
logomaker 0.8
dpks 0.1.4

For manuscripts utilizing custom algorithms or software that are central to the research but not yet described in published literature, software must be made available to editors and reviewers. We strongly encourage code deposition in a community repository (e.g. GitHub). See the Nature Portfolio guidelines for submitting code & software for further information.

## Data

Policy information about availability of data

All manuscripts must include a data availability statement. This statement should provide the following information, where applicable:

- Accession codes, unique identifiers, or web links for publicly available datasets
- A description of any restrictions on data availability
- For clinical datasets or third party data, please ensure that the statement adheres to our policy

The raw mass spectrometry data for both the porcine and human samples has been deposited to proteomeXchange under the identifier PXD048892 [https://proteomecentral.proteomexchange.org/cgi/GetDataset?ID=PXD048892]. The dataset from Van et al. was fetched from proteomeXchange with the dataset identifier PXD012210 [https://proteomecentral.proteomexchange.org/cgi/GetDataset?ID=PXD012210]. The data generated in this study are provided in the Source Data file.

## Research involving human participants, their data, or biological material

Policy information about studies with human participants or human data. See also policy information about sex, gender (identity/presentation), and sexual orientation and race, ethnicity and racism.

| | |
|---|---|
| Reporting on sex and gender | Sex and gender information was not collected in this study, thus we did not carry out any sex- or gender-based analyses. |
| Reporting on race, ethnicity, or other socially relevant groupings | Information on race, ethnicity, or other socially relevant groupings was not collected in this study, thus we did not carry out any such analyses. |
| Population characteristics | Participants were aged ≥40 years, and diagnosed with venous insufficiency with target venous leg ulcers with a total ulcer area of ≥ 40 and ≤ 120 cm^2 |
| Recruitment | Patients were identified at the Dermatology clinic's wound healing unit and contacted about potential participation in the study. |
| Ethics oversight | The use of human wound materials was approved by the Swedish ethical review authority (etikprövningsmyndigheten application number 2023-05051-02). |

Note that full information on the approval of the study protocol must also be provided in the manuscript.

# Field-specific reporting

Please select the one below that is the best fit for your research. If you are not sure, read the appropriate sections before making your selection.

☒ Life sciences ☐ Behavioural & social sciences ☐ Ecological, evolutionary & environmental sciences

For a reference copy of the document with all sections, see nature.com/documents/nr-reporting-summary-flat.pdf

# Life sciences study design

All studies must disclose on these points even when the disclosure is negative.

| | |
|---|---|
| Sample size | No statistical methods were used to predetermine sample size estimates. For the porcine and human datasets, the sample size was based on the availability of relevant samples in the biobank and was deemed sufficient for this exploratory analysis. For the porcine samples these criteria included: sufficient protein amounts, sufficient fluid amounts, no contamination from other bacteria. The selection of human samples was based on bacterial presence as determined by MALDI and enzyme activity as determined by zymography.<br><br>Puthia, M., Butrym, M., Petrlova, J., Stromdahl, A.C., Andersson, M.A. et al. A dual-action peptide-containing hydrogel targets wound infection and inflammation. Sci Transl Med 12 (2020). |
| Data exclusions | After sample selection and analysis by mass spectrometry, no data was excluded from analysis. |
| Replication | Multiple samples were generated from multiple wounds, pigs and time-points. For the human material, different patient wounds at different time-points were studied. For the training of the machine learning classifier, the model was trained using k-fold cross-validation to reduce the risk of over-fitting.<br><br>A blinded re-run of 12 samples was performed. These samples were chosen in a stratified but randomized manner from both singly infected samples and uninfected controls collected on day 1, with four samples selected from each group. The entire sample preparation pipeline and mass-spectrometry analysis were repeated but this time the sample annotations were blinded. We then analyzed the samples using the computational workflow and visualized the results using UMAPs to determine reproducibility. Since the analysis of the original samples, the mass spectrometry park has been upgraded from timsTOF pro to timsTOF HT. The replicates group with the correct infection type and time point demonstrating the replicability of the sample preparation and analysis pipeline. |

| | |
|---|---|
| Randomization | Beyond the re-run of 12 samples, no randomization was used in this study. Covariates in the original study (Puthia et al.) such as batch variation in animals was controlled by testing the different conditions in each batch. To avoid bias in selection of more or less healthy animals in groups, the groups were assigned before the experiments were started.<br><br>Puthia, M., Butrym, M., Petrlova, J., Stromdahl, A.C., Andersson, M.A. et al. A dual-action peptide-containing hydrogel targets wound infection and inflammation. Sci Transl Med 12 (2020).<br><br>For the human samples, wounds were randomized to various treatments in the original study from which the samples originated, only samples from control wounds are included in this study. |
| Blinding | Beyond the re-run of 12 samples, the experiments were not blinded. |

# Reporting for specific materials, systems and methods

We require information from authors about some types of materials, experimental systems and methods used in many studies. Here, indicate whether each material, system or method listed is relevant to your study. If you are not sure if a list item applies to your research, read the appropriate section before selecting a response.

## Materials & experimental systems

| n/a | Involved in the study | |
|---|---|---|
| ☒ | ☐ | Antibodies |
| ☒ | ☐ | Eukaryotic cell lines |
| ☒ | ☐ | Palaeontology and archaeology |
| ☐ | ☒ | Animals and other organisms |
| ☐ | ☒ | Clinical data |
| ☒ | ☐ | Dual use research of concern |
| ☒ | ☐ | Plants |

## Methods

| n/a | Involved in the study | |
|---|---|---|
| ☒ | ☐ | ChIP-seq |
| ☒ | ☐ | Flow cytometry |
| ☒ | ☐ | MRI-based neuroimaging |

## Animals and other research organisms

Policy information about studies involving animals; ARRIVE guidelines recommended for reporting animal research, and Sex and Gender in Research

| | |
|---|---|
| Laboratory animals | Female Göttingen minipigs weighing 14–16 kg, aged 6-7 months were used |
| Wild animals | No wild animal were involved in this study. |
| Reporting on sex | Gender was not considered in the data analysis. |
| Field-collected samples | Not involved. |
| Ethics oversight | All animal experiments are performed according to Swedish Animal Welfare Act SFS 1988:534 and were approved by the Animal Ethics Committee of Malmö/Lund, Sweden (permit number M131-16).All animals received care according to the USA Principles of Laboratory Animal Care of the National Society for Medical Research, Guide for the Care and Use of Laboratory Animals, National Academies Press (1996). |

Note that full information on the approval of the study protocol must also be provided in the manuscript.

## Clinical data

Policy information about clinical studies

All manuscripts should comply with the ICMJEguidelines for publication of clinical research and a completedCONSORT checklist must be included with all submissions.

| | |
|---|---|
| Clinical trial registration | NCT05378997 |
| Study protocol | The full trial protocol is not made publicly available as it contains proprietary information. |
| Data collection | Participants were recruited from 06-02-2023 to 06-03-2023 at the Dermatology clinic at Skåne University Hospital, Lund, Sweden |
| Outcomes | This study was based on biobank samples collected from control patients as an exploratory outcome in the described clinical trial. Therefore the outcomes in the clinical trial are not applicable to this study. |

## Plants

| | |
|---|---|
| Seed stocks | *Report on the source of all seed stocks or other plant material used. If applicable, state the seed stock centre and catalogue number. If plant specimens were collected from the field, describe the collection location, date and sampling procedures.* |
| Novel plant genotypes | *Describe the methods by which all novel plant genotypes were produced. This includes those generated by transgenic approaches, gene editing, chemical/radiation-based mutagenesis and hybridization. For transgenic lines, describe the transformation method, the number of independent lines analyzed and the generation upon which experiments were performed. For gene-edited lines, describe the editor used, the endogenous sequence targeted for editing, the targeting guide RNA sequence (if applicable) and how the editor was applied.* |
| Authentication | *Describe any authentication procedures for each seed stock used or novel genotype generated. Describe any experiments used to assess the effect of a mutation and, where applicable, how potential secondary effects (e.g. second site T-DNA insertions, mosiacism, off-target gene editing) were examined.* |
