# Supplementary figures and images for: Peptide clustering enhances large-scale analyses and reveals proteolytic signatures in mass spectrometry data
Source: Nat Commun. 2024 Aug 20;15:7128. doi: 10.1038/s41467-024-51589-y (PMC11336174; doi:10.1038/s41467-024-51589-y)

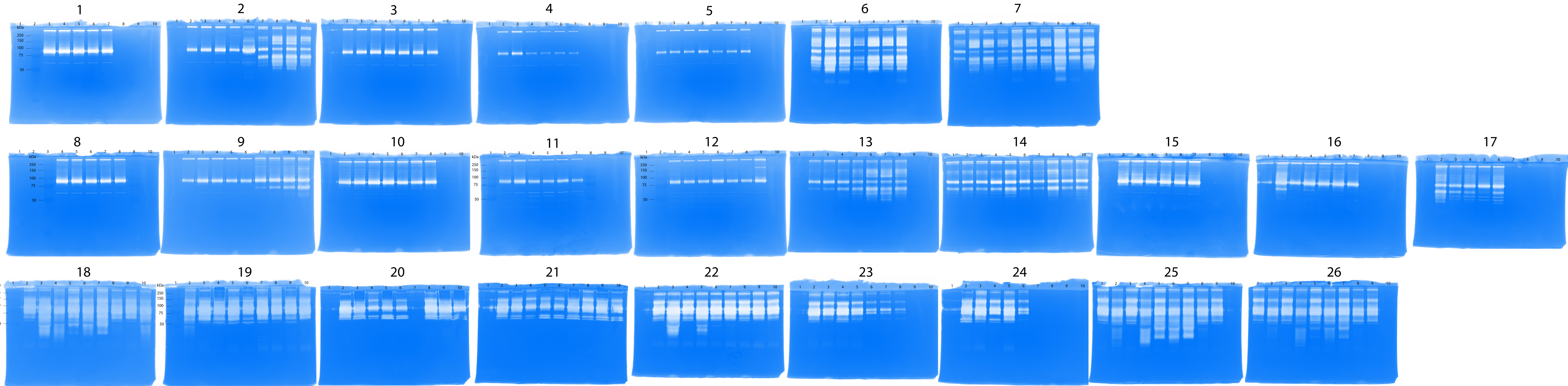

Supplement: Supplementary file 4 — Source Data [file 41467_2024_51589_MOESM4_ESM.zip › source_data/zymograms.pdf]
